# Participating in Two Video Concussion Education Programs Sequentially Improves Concussion-Reporting Intention

**DOI:** 10.1089/neur.2021.0033

**Published:** 2021-12-08

**Authors:** Daniel H. Daneshvar, Christine M. Baugh, Roberto D. Lama, Maya Yutsis, Roy D. Pea, Shelley Goldman, Gerald A. Grant, Robert C. Cantu, Lee M. Sanders, Ross D. Zafonte, Brian Hainline, Piya Sorcar

**Affiliations:** ^1^ Department of Physical Medicine and Rehabilitation, Harvard Medical School; Boston, Massachusetts, USA; Massachusetts General Hospital; Boston, Massachusetts, USA; Spaulding Rehabilitation Hospital, Boston, Massachusetts, USA.; ^2^ Chronic Traumatic Encephalopathy Center, Boston University School of Medicine, Boston, Massachusetts, USA.; ^3^ Center for Bioethics and Humanities, University of Colorado Denver Anschutz Medical Campus, Aurora, Colorado, USA; Division of General Internal Medicine, University of Colorado School of Medicine, Aurora, Colorado, USA.; ^4^ School of Engineering, Stanford University, Stanford, California, USA.; ^5^ Department of Neurology, Stanford School of Medicine, Palo Alto, California, USA.; ^6^ Graduate School of Education, Stanford University, Stanford, California, USA.; ^7^ Department of Neurosurgery, Stanford School of Medicine, Palo Alto, California, USA.; ^8^ Cantu Concussion Center, Emerson Hospital, Concord, Massachusetts, USA.; ^9^ Department of Pediatrics, Stanford School of Medicine, Palo Alto, California, USA.; ^10^ Brigham and Women's Hospital, Boston, Massachusetts, USA.; ^11^ Indiana University School of Medicine, Indianapolis, Indiana, USA; New York University Langone Medical Center, New York, New York, USA.; ^12^ Stanford Center for Innovation in Global Health, Stanford School of Medicine, Palo Alto, California, USA.

**Keywords:** concussion, education, prevention, reporting

## Abstract

Undiagnosed concussions increase the risk of additional concussion and persistent symptoms from concussion. Because there are no reliable objective markers of concussion, self-reporting of subjective and non-visible symptoms are critical to ensuring proper concussion management. For this reason, educational interventions target concussion reporting, but the majority of studies have examined the efficacy of single educational interventions or compared interventions to one another. This randomized crossover study sought to identify whether there was benefit to administering multiple concussion education programs in tandem, back to back. The study randomized 313 male high school football players to first receive CrashCourse concussion education (CC) or Centers for Disease Control and Prevention video concussion education (CDC) followed by crossover with the other education. Athlete concussion-reporting intention, attitudes, subjective norms, perceived behavioral control, and enjoyment of education were assessed at baseline and after each intervention. There were statistically significant improvements across all measures, both after single intervention and crossover (all *p* < 0.001). Secondary analyses examining differences between education found that athletes reported higher enjoyment of concussion education immediately after participating in CC, as compared to CDC (*p* < 0.001). These findings demonstrate an additive benefit to implementing CC and CDC education in tandem, without decrement in enjoyment of concussion education after experiencing dual educations; in fact, enjoyment of concussion education improved after receiving education programs back to back. These educational programs appear to complement one another, and the results support the use of multi-modal concussion education to differentially target and maximize concussion reporting.

## Introduction

An estimated 1.1 million to 1.9 million concussions occur annually in children 18 years or younger in the United States, with an estimated 0.5 million to 1.2 million of these concussions not properly diagnosed.^1,2^ These underdiagnosed concussions increase the risk of subsequent concussion or persistent symptoms from concussion, especially when an athlete continues to participate in activities with a high risk of traumatic brain injury (TBI).^3–8^ Given the increased risks of successive concussion and prolonged recovery associated with ongoing play, undiagnosed concussions pose a serious public health issue.^9^

The manifestations of concussions can often be difficult to observe or characterize, and many athletes under-report concussion symptoms and return to play.^2,4,10,11^ Proactive measures, such as concussion education and increased youth awareness, may serve to improve concussion reporting and minimize the risk of successive TBI.^12–16^ As a result, multiple concussion education programs have been created, each of which present content uniquely.

**FIG. 1.**
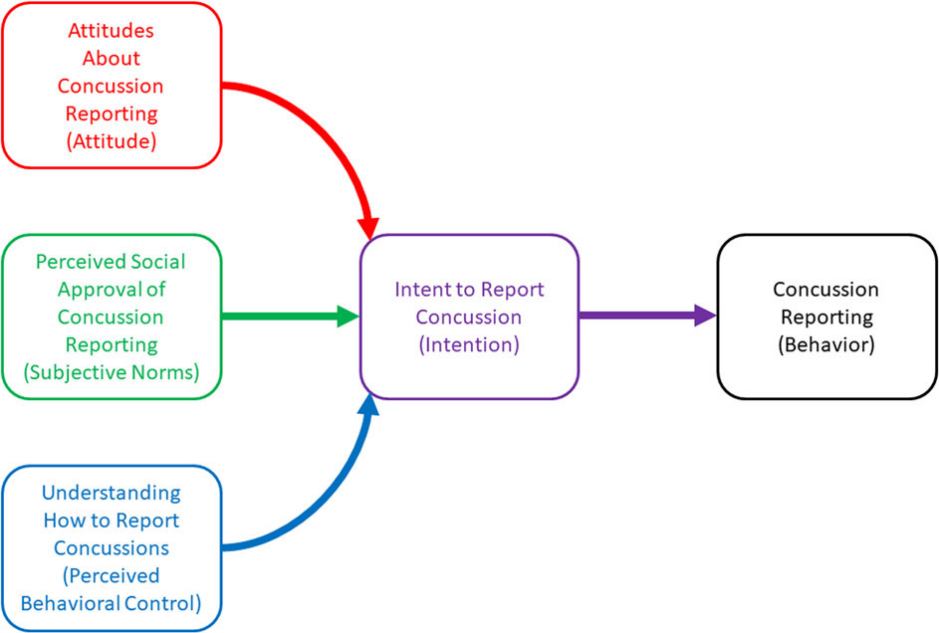
Theory of planned behavior in the context of concussion reporting.^22^

Because of the complexities associated with measuring concussion-reporting behavior change, studies have evaluated the efficacy of concussion education programs using outcomes proximally related to behaviors, by either examining effects on concussion knowledge, or constructs derived from the theory of planned behavior (TPB; Fig. 1).^12,13,15,17–21^ TPB, first described by Ajzen in 1991, aims to identify the factors associated with behavior change. TPB posits that behavioral changes are signaled by behavioral intention (motivation), which is directly influenced by behavioral beliefs (attitudes about reporting), normative beliefs (subjective and social approval of reporting), and perceived behavioral control (understanding how to report).^22^

Over the past 30 years, TPB has been tested in a variety of contexts.^23^ Previous work demonstrates that TPB can be a useful predictive model for adolescent concussion-reporting behavior, with all three constructs (attitudes, subjective norms, and perceived behavioral control) associated with concussion-reporting intention (motivation) and concussion reporting (behavior change).^12,13,20,24,25^ Few studies have directly compared the efficacy of different education programs; these studies demonstrated that athletes who experience different concussion education programs have variable improvement in concussion-reporting intention, concussion attitudes, subjective norms, and perceived behavioral control.^13,26^

There may, however, be benefits to administering multiple concussion education programs to improve concussion reporting.^15^ Each educational intervention has a different format with different foci and, as a result, may have different strengths in influencing constructs related to the reporting decision. The benefit of administering multiple educational interventions has not been previously assessed. A common concern with implementing multiple interventions is that the learner may be overburdened, resulting in less satisfaction with an educational program, and thus less compliance.^27^ Additionally, different concussion education programs may be preferentially enjoyed by different learners; there is evidence that concussion education enjoyment may have a moderating effect on concussion reporting.^28^

Over the past 10 years, the most commonly used concussion education resources in the United States have been created and provided by the Centers for Disease Control and Prevention (CDC). In 2010, the CDC partnered with the National Federation of State High School Associations (NFHS) to release the free “Concussion in Sports” course, which has been viewed >2.2 million times.^29^

Since launching in September 2018, the CrashCourse (CC) concussion education has been widely adopted, including by the majority of U.S. youth football organizations (e.g., U.S. Football, American Youth Football and Pop Warner), various U.S. Olympic Committees (e.g., U.S. Speedskating, U.S. Field Hockey, and U.S. Wrestling), medical organizations (e.g., Brain Injury Association of America, Lucille Packard Children's Hospital Stanford), and several U.S. high schools (e.g., state high school athletic associations of North Carolina and Arkansas).^30^ A recent randomized controlled trial demonstrated that athletes randomized to CC reported greater intent to report concussion, more knowledge, and improved concussion-reporting attitudes when compared to CDC video and written materials.^13^

The present study sought to evaluate the potential benefits of receiving dual concussion education programs, with persons randomized to first receive either CDC or CC, followed by crossover with the other education program. We hypothesized that athletes would improve in concussion-reporting intention, attitudes, subjective norms, perceived behavioral control, and enjoyment of education, after one educational intervention, with significantly greater gains after the second educational program. Secondary analyses were performed to evaluate differences in outcomes between the two programs.

## Methods

### Participants

**FIG. 2.**
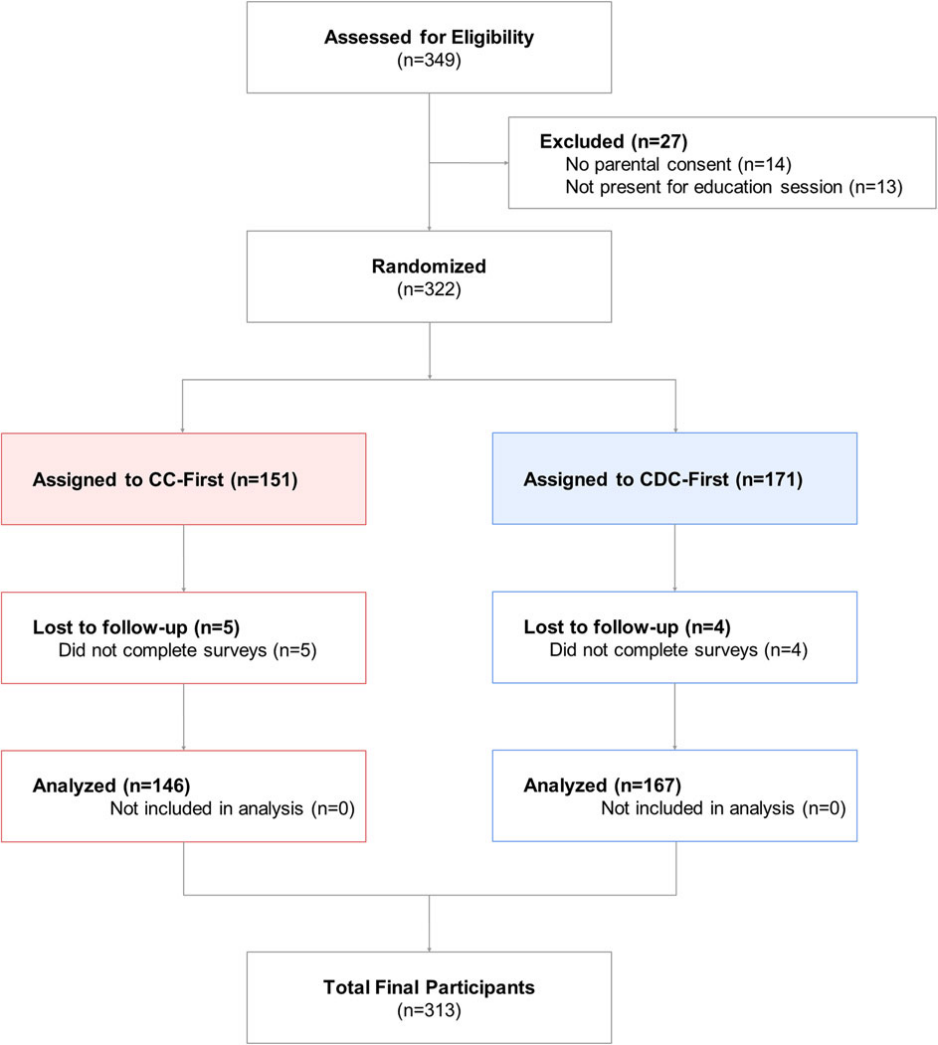
Study enrollment and randomization. CC, CrashCourse concussion education; CDC, Centers for Disease Control and Prevention video-based concussion education.

Participants were male athletes from seven high school football teams in Colorado. These schools were varied in terms of racial composition (minority enrollment ranging from 18% to 94%; mean, 38%) and socioeconomic status (percent of students qualifying for free lunch ranging from 8% to 86%; mean, 26%). Participants were included if they were in attendance for practice on the study date and provided signed assent and parental consent. Participants were excluded if they did not complete >50% of the questionnaires. In total, 349 participants were screened for inclusion; 14 did not provide a signed parental consent and 13 were absent on the initial study date. Nine athletes were excluded because of incomplete participation. Figure 2 outlines study enrollment. Participation in this research study was entirely voluntary and not a pre-requisite for football participation to meet concussion education guidelines. All research activities were reviewed and approved by the Stanford Medicine Institutional Review Board.

### Design

All participants (*n* = 313) completed study questionnaires and education on individual computers in July 2019. Participants first completed baseline study questionnaires before being randomized with a computer-based random number generator to be assigned to one of two groups: CC-First or CDC-First. CC-First (*n* = 146) received the CC concussion education curriculum followed by the CDC/NFHS “Concussion in Sports” video curricula. CDC-First (*n* = 167) received the CDC curriculum followed by CC. All athletes completed a post-education questionnaire immediately after exposure to the first educational curricula and another questionnaire immediately after exposure to the second educational curricula. Two research team members were present for the entirety of the administration to monitor students and were provided guidelines with escalating actions in case of disruptive behavior.

### Interventions

#### CrashCourse concussion education

In September 2018, the 501(c)3 non-profit organization, TeachAids, released a free video-based concussion education program. CC was created with a user-centered formative design process by iteratively incorporating feedback from hundreds of athletes, coaches, athletic trainers, education researchers, and scientific experts. The resulting 12-min video places the learner in a first-person game situation, where they experience a concussion and are presented with the choice to “Stay in the Game” or “Take a Knee” and be evaluated. The consequences of the learner's decision are further explored. Subsequently, the consequences of the alternate decision are shown, ensuring that learners are provided all information. Learners are taught to recognize concussion signs and symptoms, understand the importance of reporting, and seek medical attention for diagnosis and management. This narrative-driven interactive program is guided by near-peer Division I collegiate football players.^30^

#### Centers for Disease Control and Prevention /National Federation of State High School Associations “Concussion in Sports” video

The CDC and NFHS partnered in 2010 to release the free “Concussion in Sports” online course, which was subsequently revised in 2018.^29^ The content is geared toward student-athletes, parents, and coaches. These materials engage learners by incorporating 25 min of videos and six slides, followed by nine questions, to teach learners concussion signs and symptoms, highlight the effects of sports-related concussion, and provides protocols to manage a suspected concussion including how to help players return to play safely after a concussion. The course is hosted by Michael Koester, MD, chair of the NFHS Sports Medicine Advisory Committee.^31^

### Outcomes

All outcomes, except perceived behavioral control, were assessed based on athlete responses to questions on a 5-point Likert scale, from 1 = “strongly disagree” to 5 = “strongly agree,” using TPB-informed questions informed by past work.^22,24,32,33^ Each question was individually chosen to ensure construct validity while maximizing assessment brevity. When multiple questions were asked related to a single construct, internal consistency was assessed, with Cronbach's alpha reported, and scores were averaged. The text of the specific questions asked of each athlete are below.

#### Concussion-reporting intention

Athletes were asked to rate, “If I think I may have a concussion, I intend to report it to a medical professional (doctor, athletic trainer, nurse)” and “if my teammates think they may have a concussion, they would report it to a medical professional (doctor, athletic trainer, nurse)” (Cronbach's alpha = 0.86).

#### Concussion-reporting attitudes

Athletes were asked to rate, “I understand the importance of telling someone if I suspect I have a concussion.”

#### Subjective norms

Athletes were asked to rate, “If I report what I suspect might be a concussion, my teammates would think I made the right decision”; “if I report what I suspect might be a concussion, my parents would think I made the right decision”; and “if I report what I suspect might be a concussion, my coaches would think I made the right decision” (Cronbach's alpha = 0.88).

#### Perceived behavioral control

Perceived behavioral control was assessed based on athlete responses to three questions on a 100-point scale, from 1 = “cannot do” to 100 = “definitely can do,” adapted from previous work.^34^ Specifically, athletes were asked to rate whether, “I can identify when I have a concussion”; “I can immediately tell a medical professional that I have a concussion”; and “I can eventually tell a medical professional that I have a concussion.” Scores were averaged to create a composite score (Cronbach's alpha = 0.89).

#### Enjoyment of education

Athletes were asked to rate, “I am satisfied with the concussion education I have received” and “I enjoyed the concussion education I have received” (Cronbach's alpha = 0.76).

### Statistical analysis

Independent-samples *t*-tests (continuous measures) or chi-square (categorical measures) were used to assess demographic differences between athletes randomized to CC-First and CDC-First. Fisher's exact test was used in place of chi-square when expected cell values were <5. For primary and secondary outcomes, repeated measures analysis of variance (ANOVA), with one between-subjects factor (randomization group) and one within-subject factor (time points: i.e., pretest, following first education, and following second education). *Post hoc* testing examined pair-wise differences between time points. Secondary analyses via repeated-measures ANOVA examined differences between education formats by including one between-subjects factor (education type) and one within-subject factor (time point). When sphericity could not be assumed, Greenhouse-Geisser corrections were applied. Although these analyses assume normality, an advantage of repeated measures ANOVA is that it is robust to violations of normality, particularly with sample sizes >20. To account for multiple comparisons, false discovery rate p-values were calculated based on the Benjamini-Hochberg procedure and are presented for analyses of all primary or secondary outcomes. Analyses were performed using IBM SPSS Statistics (Version 20.0; IBM Corp., Armonk, NY) or R software (Version 3.6; R Foundation for Statistical Computing).

## Results

Demographic information is provided in Table 1. Athletes were 13.1% freshman, 28.3% sophomore, 26.9% juniors, and 31.7% seniors (of note, in the United States, freshmen typically begin high school at ages 14–15 and are seniors at ages 17–18). They started playing football at an average age of 9.4 ± 3.0 years and had played football for an average of 6.5 ± 3.1 years. There were no significant differences in grade, football position, duration of football career, or age of starting football between athletes randomized to receive CC-First and CDC-First. Please see the Supplementary Materials for demographic differences in baseline measures.

**Table 1.** Demographics and Characteristics of Study Participants

	Education format			
	CC-First (*n* = 145*^a^*)	CDC-First* (n* = 167)	Overall (*n* = 312)	p value^b^
Grade (%)				0.40
Freshman	19 (13.1)	32 (19.2)	51 (16.3)	
Sophomore	41 (28.3)	51 (30.5)	92 (29.5)	
Junior	39 (26.9)	37 (22.2)	76 (24.4)	
Senior	46 (31.7)	47 (28.1)	93 (29.8)	
Position (%)				0.58
Defensive back	16 (11.0)	27 (16.2)	43 (13.8)	
Defensive line	21 (14.5)	21 (12.6)	42 (13.5)	
Fullback	1 (0.7)	2 (1.2)	3 (1.0)	
Kicker	2 (1.4)	0 (0.0)	2 (0.6)	
Linebacker	23 (15.9)	26 (15.6)	49 (15.7)	
Offensive line	24 (16.6)	35 (21.0)	59 (18.9)	
Quarterback	8 (5.5)	8 (4.8)	16 (5.1)	
Running back	15 (10.3)	15 (9.0)	30 (9.6)	
Tight end	2 (1.4)	5 (3.0)	7 (2.2)	
Wide receiver	33 (22.8)	28 (16.8)	61 (19.6)	
Years of football	6.5 ± 3.1	5.9 ± 3.2	6.1 ± 3.2	0.07
Age started football	9.4 ± 3.0	9.8 ± 3.0	9.6 ± 3.0	0.36

^a^ One participant randomized to receive CC-First did not complete demographic information.

^b^ Independent-samples *t*-tests for continuous data, or chi-square analyses for categorical data, were used to assess demographic differences between groups.

CC, CrashCourse concussion education; CDC, Centers for Disease Control and Prevention video materials.

**Table 2.** The Relationship between Concussion Education Administered at Each Time Point and Primary and Secondary Outcomes

	All athletes (*n* = 313)	CC-First (*n* = 146)	CDC-First (*n* = 167)	p value^a^
Concussion-reporting intention				0.05
Baseline (95% CI)	4.0 (3.9, 4.1)	4.0 (3.8, 4.1)	4.1 (4.0, 4.2)	
After first education (95% CI)	4.4 (4.3, 4.5)	4.4 (4.3, 4.5)	4.4 (4.3, 4.5)	
After second education (95% CI)	4.6 (4.5, 4.7)	4.6 (4.5, 4.7)	4.5 (4.5, 4.6)	
*p* value across all time points^b^	**<0.001**	**<0.001**	**<0.001**	
*p* value baseline to first education	**<0.001**	**<0.001**	**<0.001**	
*p* value first to second education	**<0.001**	**<0.001**	**<0.001**	
Concussion attitudes				0.53
Baseline (95% CI)	4.4 (4.3, 4.5)	4.4 (4.3, 4.5)	4.4 (4.3, 4.5)	
After first education (95% CI)	4.6 (4.6, 4.7)	4.7 (4.6, 4.7)	4.6 (4.5, 4.7)	
After second education (95% CI)	4.7 (4.7, 4.8)	4.7 (4.6, 4.8)	4.7 (4.6, 4.8)	
*p* value across all time points^b^	**<0.001**	**<0.001**	**<0.001**	
*p* value baseline to first education	**<0.001**	**0.001**	**<0.001**	
*p* value first to second education	**<0.001**	**<0.001**	**<0.001**	
Concussion-reporting norms				0.53
Baseline (95% CI)	4.3 (4.2, 4.4)	4.3 (4.2, 4.4)	4.3 (4.2, 4.4)	
After first education (95% CI)	4.5 (4.5, 4.6)	4.6 (4.5, 4.6)	4.5 (4.4, 4.6)	
After second education (95% CI)	4.7 (4.6, 4.7)	4.7 (4.6, 4.8)	4.6 (4.5, 4.7)	
*p* value across all time points^b^	**<0.001**	**<0.001**	**<0.001**	
*p* value baseline to first education	**<0.001**	**<0.001**	**<0.001**	
*p* value first to second education	**<0.001**	**<0.001**	**<0.001**	
Concussion perceived behavioral control				0.53
Baseline (95% CI)	68.9 (66.6, 71.0)	69.8 (66.6, 73.0)	67.9 (64.8, 70.9)	
After first education (95% CI)	79.9 (77.9, 81.9)	80.4 (77.5, 83.3)	79.5 (76.8, 82.2)	
After second education (95% CI)	85.4 (83.6, 87.2)	86.6 (84.0, 89.2)	84.3 (81.8, 86.7)	
*p* value across all time points^b^	**<0.001**	**<0.001**	**<0.001**	
*p* value baseline to first education	**<0.001**	**<0.001**	**<0.001**	
*p* value first to second education	**<0.001**	**<0.001**	**<0.001**	
Concussion-education enjoyment				0.01
Baseline (95% CI)	3.5 (3.4, 3.6)	3.5 (3.3, 3.6)	3.5 (3.4, 3.6)	
After first education (95% CI)	4.3 (4.3, 4.4)	4.4 (4.3, 4.5)	4.2 (4.1, 4.3)	
After second education (95% CI)	4.6 (4.5, 4.6)	4.6 (4.5, 4.7)	4.6 (4.5, 4.7)	
*p* value across all time points^b^	**<0.001**	**<0.001**	**<0.001**	
*p* value baseline to first education	**<0.001**	**<0.001**	**<0.001**	
*p* value first to second education	**<0.001**	**<0.001**	**<0.001**	

False discovery rate *p* values were calculated based on the Benjamini-Hochberg procedure to account for multiple comparisons. *p* values in bold indicate significant differences.

^a^ Represents results of repeated-measures ANOVA with one between-subjects factor (the outcome being evaluated) and one within-subject factor (time point).

^b^ Represents the results of *post hoc* ANOVA.

CI, confidence interval; ANOVA, analysis of variance.

### Concussion-reporting intention

**FIG. 3.**
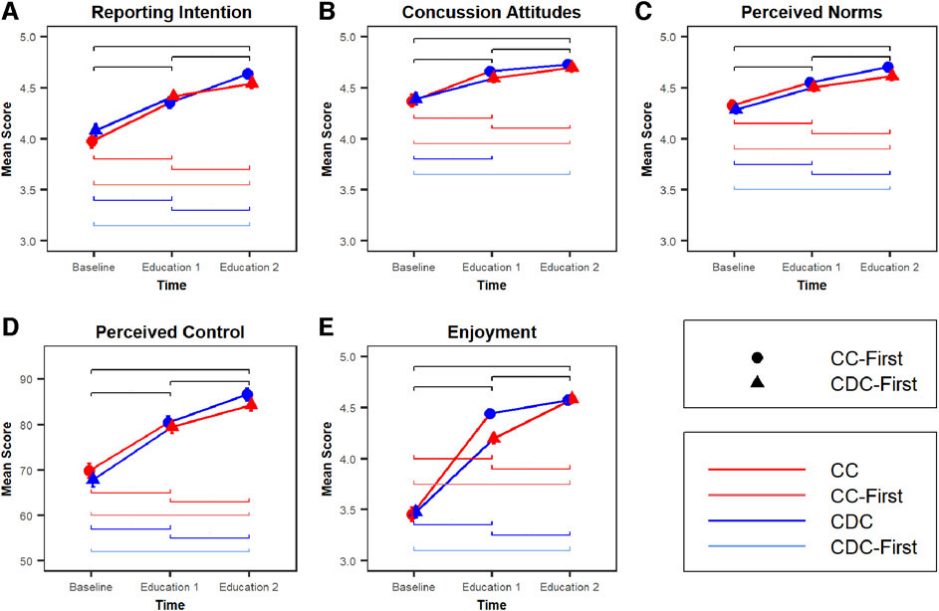
Changes in proxies of concussion-reporting intention after each educational intervention. Significance bars indicate significant differences for: all participants (black), the group randomized to receive CC-First (light red), the group randomized to receive CDC-First (light blue), immediately before and after each CC intervention (dark red), and immediately before and after each CDC intervention (blue). Error bars indicate 95% confidence intervals at each point. CC, CrashCourse concussion education; CDC, Centers for Disease Control and Prevention video-based concussion education.

Athlete concussion-reporting intention significantly improved (Fig. 3A; omnibus, *p* < 0.001), from 4.0 ± 0.8 to 4.4 ± 0.6, after the first educational intervention (*p* < 0.001) and significantly improved again to 4.6 ± 0.6 after the second educational intervention (*p* < 0.001). There was no difference between concussion education administered (*p* = 0.05).

### Concussion-reporting attitudes

Attitudes about concussion reporting significantly improved (Fig. 3B; omnibus, *p* < 0.001) from 4.4 ± 0.7 to 4.6 ± 0.5 after the first educational intervention (*p* < 0.001) and significantly improved again to 4.7 ± 0.5 after the second educational intervention (*p* < 0.001). There was no difference between concussion education administered (*p* = 0.53).

### Subjective norms

Athlete subjective norms related to concussion reporting significantly improved (Fig. 3C; omnibus, *p* < 0.001), from 4.3 ± 0.6 to 4.5 ± 0.5, after the first educational intervention (*p* < 0.001) and significantly improved again to 4.7 ± 0.5 after the second educational intervention (*p* < 0.001). There was no difference between concussion education administered (*p* = 0.53).

### Perceived behavioral control

Athlete perceived behavioral control about concussion reporting significantly improved (Fig. 3D; omnibus, *p* < 0.001), from 68.8 ± 19.9 to 79.9 ± 17.7, after the first educational intervention (*p* < 0.001) and significantly improved again to 85.4 ± 16.2 after the second educational intervention (*p* < 0.001). There was no difference between concussion education administered (*p* = 0.53).

### Enjoyment of education

Athlete enjoyment of concussion education significantly improved (Fig. 3E; omnibus, *p* < 0.001), from 3.5 ± 0.8 to 4.3 ± 0.6, after the first educational intervention (*p* < 0.001) and significantly improved again to 4.6 ± 0.6 after the second educational intervention (*p* < 0.001). There were significant differences by education administered and time (*p* = 0.01), with athletes reporting enjoyment of 4.5 ± 0.5 immediately after receiving CC and athletes reporting enjoyment of 4.4 ± 0.6 immediately after receiving CDC.

## Discussion

This present study found that all measured concussion-related outcomes improved after both concussion programs and were further improved after a second educational module. There were also improvements in TPB constructs proximally related to concussion reporting, namely concussion-reporting ntention, concussion attitudes, perceived norms, and perceived behavioral control. These changes occurred regardless of education administered. Importantly, there were additional increases associated with administering a second education. This study was uniquely able to assess these changes because of its randomized crossover design, ensuring that athletes could serve as their own controls, while they all received both educational interventions.

There are several explanations for why providing different educational interventions in tandem provide additive benefit. There may be a benefit to reinforcing ideas, especially because repeated messaging from different sources lends further credibility to the message. Also, because each intervention was created to be as parsimonious as possible, there are ideas emphasized in one education that may have been left out of the other, often because of time constraints. Experiencing multiple programs allows the learner to have a more comprehensive education about the importance of concussion reporting.

There has been concern that learners would experience fatigue when experiencing prolonged education,^27^ or that additional education may result in a resistance to the desired behavior.^35^ However, this study demonstrates that the benefits associated with providing multiple education programs do not come at the expense of athlete enjoyment; athletes actually reported significant increases in their enjoyment and satisfaction with the concussion education programs that they had received, even after receiving the second of two educational interventions back to back.

The present study analyzed the effect of these educational programs sequentially, back to back. However, there may be benefits to intervening at different time points throughout a season.^15^ For example, a more in-depth education may be best suited for the beginning of a season, with refreshers administered periodically throughout the season. Now that the benefit of multiple educational interventions has been demonstrated generally, further work can explore optimal timing.

Interestingly, the only outcome that significantly differed between education types was enjoyment of the concussion education received. The lack of other differences between CC and CDC educational materials differs from previous studies evaluating these programs.^13^ This discrepancy may be related to the lack of longitudinal follow-up in the present study. Specifically, previous work randomized athletes to receive an educational intervention and evaluated for sustained improvements in concussion-reporting–related outcomes at 1 month. Although the crossover design in the present study allowed all athletes to serve as their own control, the extent to which improvements persisted over time could not be assessed because all athletes received both educational interventions. However, the observation that athletes exhibited a relative preference to CC over CDC is relevant given that research in other fields has demonstrated a relationship between enjoyment and behavior change.^36–39^

Additionally, recent work has demonstrated a moderating effect of educational enjoyment on concussion-reporting intention.^28^ Because every state mandates that its athletes receive concussion education, learners may be less excited about participation in the education.^40^ The role of educational enjoyment is likely an important factor in ensuring engagement and maximizing learning.^28,41,42^

There are several limitations to the present study, in addition to the aforementioned inability to track retention of changes inherent to the crossover design. The present study only measured constructs related to TPB, rather than true behavior change. Although changes in concussion-reporting intention has been estimated to account for 20% of concussion-reporting behavior,^24^ the clinical relevance of a specific change in intention to report concussions, assessed with a Likert scale, is uncertain. However, given estimates of >1 million unreported concussions,^1,2,4^ and the risk of prolonged recovery associated with improperly managed injuries,^3,5,8,9^ even a small improvement in concussion reporting would yield significant public health benefits. Participants may have also responded in a manner that they perceive is “preferred,” but their responses may not reflect true concussion-reporting intention.

Additionally, all participants were recruited from schools in a single region in the United States; this lack of geographical diversity may limit applicability given that athletic culture, attitudes, and beliefs exhibit significant regional variation. Additionally, all the athletes were male, and were football players, further limiting generalizability beyond these groups. Also, the number of demographical questions asked was limited, because the study required all participants to complete two concussion education programs (both CC and CDC) and three questionnaires (baseline, post-education 1, and post-education 2) in a single session, meaning that additional group differences could not be explored. As such, differences based on race, socioeconomic status, and other factors potentially relevant to concussion reporting could not be explored. However, the schools that athletes were recruited from represent a wide range of racial and socioeconomic diversity. Finally, both educational programs were video-based interventions; it is likely that mixing educational formats would have different results.

## Conclusion

This study showed that implementing two different educational programs in tandem results in greater improvements in TPB constructs related to concussion reporting, as compared to a single education alone. Additionally, contrary to concerns of fatigue after participating in multiple educational interventions, athletes reported greater enjoyment and satisfaction with the concussion education they received after both interventions in tandem. Given the largely invisible nature of concussion manifestation and the important role of athlete reports in concussion identification, concussion education plays an important role in improving concussion self-reporting. Future studies evaluating the optimal timing, setting, duration, and other contextual factors for repeat concussion education are warranted.

## Supplementary Material

Supplemental data

## Abbreviations Used

ANOVA: analysis of variance
CC: CrashCourse
CDC: Centers for Disease Control and Prevention
NFHS: National Federation of State High School Associations
TBI: traumatic brain injury
TPB: theory of planned behavior
