# Supplementary material for: Participating in Two Video Concussion Education Programs Sequentially Improves Concussion-Reporting Intention
Source: Neurotrauma Rep. 2021 Dec 8;2(1):581–91. doi: 10.1089/neur.2021.0033 (PMC8742279; doi:10.1089/neur.2021.0033)
Supplement: Supplemental data [file Supp_Data.zip › Daneshvar et al Colorado Crossover Supplement 101521.docx]

**Supplementary Materials**

**Table of Contents**

Methods: *Statistical methods for demographic analyses* . . . . 2

Results: *Demographic Differences in Baseline Measures* . . . . 2

**Methods**

*Statistical methods for demographic analyses*

To determine the relationship between baseline measures and demographic measures (specifically duration of play, age of starting football, and grade level), ordinal logistic regressions were used for Likert outcomes, and linear regression for continuous outcomes, Simple ANOVA was used to examine differences in outcomes between schools.

**Results**

*Demographic Differences in Baseline Measures*

There was decreased baseline concussion reporting intention for athletes who had played football for more years (p=0.02; OR=0.90, 95%CI=0.85-0.96) and who were in a higher grade (p=0.005, OR=0.71, 95%CI=0.59-0.86). However, there was no relationship between age of starting football and baseline concussion reporting intention (p=0.12), attitudes (p=0.72), satisfaction (p=0.23), subjective norms (p=0.17), or self-efficacy (p=0.58). We also found no relationship between duration of football and baseline attitudes (p=0.22), satisfaction (p=0.52), subjective norms (p=0.15), or self-efficacy (p=0.72). We also found no relationship between grade level and attitudes (p=0.17), satisfaction (p=0.11), subjective norms (p=0.11), or self-efficacy (p=0.96). There were no differences between schools on baseline concussion reporting intention (p=0.05), attitudes (p=0.25), satisfaction (p=0.45), subjective norms (p=0.65), or self-efficacy (p=0.63).
