# Supplementary material for: Participating in Two Video Concussion Education Programs Sequentially Improves Concussion-Reporting Intention
Source: Neurotrauma Rep. 2021 Dec 8;2(1):581–91. doi: 10.1089/neur.2021.0033 (PMC8742279; doi:10.1089/neur.2021.0033)
Supplement: Supplemental data [file Supp_Data.zip › Colorado Studies Manual.docx]

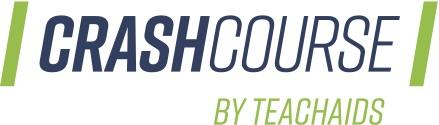


2019 Colorado Studies Manual

In July 2019, TeachAids administered a 45-60 minute survey to high school football players across 7 high schools in Colorado to test the efficacy of CrashCourse Football.

The survey was composed of the interactive CrashCourse football educational curricula, the CDC’s Concussion in Sports curricula, and three sets of behavior and self-efficacy questions. We chose the CDC’s educational videos because they are seen as the current “standard” for concussion education, and supply material for many coaching certification courses.

The structure of the survey was as follows: questions, curriculum 1, questions, curriculum 2, questions. Each student watched both CrashCourse and CDC curricula, but they were randomized into two groups: one group watched CrashCourse first, the other watched CDC first. In this way, we sought to find whether CrashCourse elicits positive change in behavior and self-efficacy at an equal or higher rate as CDC material.

**Research Assistants**

Angelina Lo (909-438-5292, [alo13@alumni.stanford.edu](mailto:alo13@alumni.stanford.edu))

Lea Tenekedjieva (919-949-2938, [lten@alumni.stanford.edu](mailto:lten@alumni.stanford.edu))

**Manual Authors**

Angelina Lo (909-438-5292, [alo13@alumni.stanford.edu](mailto:alo13@alumni.stanford.edu))

Lea Tenekedjieva (919-949-2938, [lten@alumni.stanford.edu](mailto:lten@alumni.stanford.edu))

**Last updated**: 11/27/19 by Lea Tenekedjieva (919-949-2938, [lten@alumni.stanford.edu](mailto:lten@alumni.stanford.edu))

[IRB](#_flgoryr7xm6m)

[School Recruitment](#_qt9lgfg3ow03)

[Booking Studies](#_4zqnwj1jn043)

[Connecting with Schools and Coaches](#_pbktkucqyfo)

[Qualtrics Surveys](#_cjylr6gmq1fz)

[Anticipating technical issues](#_hxcsxqtgi71y)

[Travel Details](#_q418gst9maoa)

[Booking](#_exslqqe4h21b)

[Packing List](#_fzu6tlsiym9v)

[Upon Arrival - Buy](#_s8tw05p0mzxq)

[Upon Arrival - Print](#_oyuwweeruqde)

[Preparing for Each School](#_5oyefsqu3yuv)

[Randomization Sheet](#_autx8w2gpi83)

[To bring to each school](#_34rqyfbw6o2)

[At the schools](#_hv6y1fddgdzt)

[When Students begin Arriving](#_lmuvrptj1c9s)

[Conducting the Study](#_ndiyzgppx5em)

[Script](#_xwhhtfvgo9ux)

[Action items while students are taking study](#_3dkxinmgwj6x)

[If participants are being disruptive](#_cau6a5k9hoss)

[Troubleshooting tech issues](#_9g4p3clz674l)

[After Study](#_h9cy3yyxdde8)

[Lessons Learned](#_99k9b05g9juo)

##

## IRB

**Key Advisors**

Piya Sorcar ([piya@teachaids.org](mailto:piya@teachaids.org))

Maya Yutsis ([myutsis@stanfordhealthcare.org](mailto:myutsis@stanfordhealthcare.org); for sharing Google documents: [yutsis.maya@gmail.com](mailto:yutsis.maya@gmail.com))

Daniel Daneshvar ([daneshvar@alum.mit.edu](mailto:daneshvar@alum.mit.edu); for sharing Google documents: [dhdaneshvar@gmail.com](mailto:dhdaneshvar@gmail.com) or dan@teachaids.org)

**IRB Writing Process**

Because our Protocol Director (Maya Yutsis) is a member of the medical school, we were required to submit a medical IRB. However, we wrote “Not applicable” for many sections of the IRB and were not required to file a HIPAA document since we are not running a medical study.

Note: One of the most unexpectedly difficult parts of the IRB process is that we were required to submit a signed letter of agreement for each school before we were allowed to do work in the school. If possible, it’s best to ask a district-level administrator to sign off on a letter of agreement so that you are covered for all schools in the area. The IRB allowed us to submit a letter of agreement from the District Athletic Director on behalf of all the schools in the district, as long as we’ve had some form of communication/confirmation from the school as well.

**Application Timeline**

Application begun: mid-May

Application submitted: May 31st

Sent back for edits/comments: June 14th

Edited application submitted: June 17th

Modification 1 (change parental consent form) submitted: June 29th

Modification 1 approved: July 3rd

Modification 2 (add schools) submitted: July 3rd

Modification 2 approved: July 8th

Study began: July 10th

##

## School Recruitment

We chose to run the study in Colorado because of a number of reasons. First, we realized that states on the coasts of America typically have unique cultures, and we wanted to ensure that we weren’t getting any potential biased results from these areas. Second, we recently entered into a partnership with USA Football, and they offered to put us in contact with district athletic directors with whom they had good working relationships. Since USA Football suggested 3 districts in the same area, we went ahead with that area.

Piya and Dick met with the three athletic directors in Colorado (while on another work trip) on **June 10th**. School recruitment continued throughout the following month.

**USA Football Contact**

@ (Director of Scholastic Partnerships, [epassino@usafootball.com](mailto:epassino@usafootball.com))

Other potential districts:

Tulsa (OK)

Shelby (TN)

Atlanta (GA)

Jefferson (KY)

Milwaulkee (WI)

Des Moines (IA)

Fort Wayne (IN)

Boise (ID)

Kansas City (KS)

### Booking Studies

The biggest things to consider when confirming with a school and choosing a date/time to run a study:

- Students must bring parental consent forms in order to participate. These forms should be distributed by coaches *at least* a few days before the survey, so the session should **not be run within the first few days after the team gets a break from practice.**
- Sessions should be **before, during, or after a practice.** When there is no practice scheduled, athletes will not go out of their way to come to the school.
- We must have access to **classrooms** and **wi-fi enabled computers/laptops** for the study.
  - One seat and one computer per student.
  - It is preferable to have each study condition in a different classroom (or if necessary, each condition in a different section of the computer lab/library).
  - Note: Many schools in Colorado (maybe elsewhere as well) are transitioning to giving Chromebooks for each student to use at school and at home. At one of our studies, the students forgot their laptops or brought in laptops that were not fully charged. We had to scrap a few people’s results because the laptops died towards the end of the survey. When running a study at a school using personal laptops, make sure to ask what brand of laptop the students get and purchase a few extra chargers to distribute to students if necessary.
- Research Assistants must come in **45 minutes before** the study is scheduled to take place. The coach or an affiliate must be present to let us into the rooms, address any tech challenges, etc.
- We needed at least an hour to clean-up, collect miscellaneous study items after the study, and

Note: Over the summer, study scheduling may get a little tricky. Students may be on vacation (this year, all coaches said they didn’t have practice on July 1-5), or computer labs may be closed down for software updates.

If they do not have enough resources for each student to have a seat/computer at one time, we can also run back-to-back studies.

### Connecting with Schools and Coaches

Remote communication with coaches was often difficult, as they check their emails very rarely and (understandably) do not answer phone calls from unknown, out-of-state numbers. What we’ve found most effective is to ‘cc’ school or district administrators into our emails, as they often check their emails more frequently and are able to contact the coaches for us.

Once the coach has come onboard and agreed to run the study, it’s best to **ask for their cell phone number and communicate by text**. Even if something must be sent by email, it is best to send a follow up text telling the coach that you have emailed them.

A few weeks before the scheduled study, it is important to get in touch with the coach to **ask them how many participants they anticipate,** and remind them about **classroom reservations** and **parental consent forms**. A digital version of the consent form should be sent by email **AND** RAs should offer to drop of consent forms at the school. Ask coaches to **collect forms during each practice** so that athletes have multiple chances to bring the forms in. Having forms collected in advance also makes it easier for RAs to check in students.

If possible, it is best to **meet up with the coach a week before the study** not only to drop off consent forms, but to also put a friendly face to name. It is also easier to talk through details in person.

**Communication templates & timeline:**

**2 weeks before:** Ask coach how many students they anticipate, and remind them of the need for classroom/computer reservation. Send them a digital copy of the permission slips and offer to drop off physical copies to distribute to the kids. [[email template](https://docs.google.com/document/d/1WRwNrOr2r4yUXagJzWmqopMB9IbsQIbFCL1jZMfZO7U/edit) - also send a follow up text]

**1 week before:** Go to school to drop off permission slips, get layout of school, talk through details with coach. Remind them to collect forms during each practice. Make sure to know where RAs should meet the coach for the day of the study.

**1 day before:** Text coach to remind them the time that you’ll be arriving at school, and make sure that they (or another school contact) will be able to meet you.

## Qualtrics Surveys

Note: It’s important that multiple people click through and vet the survey and all associated educational videos (**preferably on the school district’s wifi**).

**Types of Surveys created:**

- **Vimeo Surveys:** the main type of survey used. All videos were embedded through Vimeo.
- **YouTube Surveys**: All videos were embedded through YouTube. Only 3 students used these links, due to YouTube not being accessible in some schools.
- **Testing Surveys:** created solely for the purpose of testing the surveys (no timer between videos, so you can click through the survey).

**If using the** [**surveys that Lea created**](https://drive.google.com/drive/folders/109bFeQowBRNzidVEt0rUSQrZqiZlbROa)**:**

- Remember to create a new tiny-url for the survey - this will make it much easier for the kids to type in and begin the survey. For convenience, the tinyurl should include the color that is featured on the first page.
  - https://tinyurl.com/study-blue-v Vimeo survey, Group A (CrashCourse first)
  - https://tinyurl.com/study-orange-v Vimeo survey, Group B (CDC first)
  - https://tinyurl.com/study-blue-y YouTube survey, Group A (CrashCourse first)
  - https://tinyurl.com/study-orange-y YouTube survey, Group A (CrashCourse first)

**If creating a survey from scratch:**

Creating a survey on Qualtrics is a lengthy process that requires lots of care and attention to detail. You can access the Qualtrics files that we used for this study [here](https://drive.google.com/drive/u/1/folders/1-w1EFlsH9tpyAso_EWE1XTquXs0uI0Gc). Anyone can import the .qsf files to their Qualtrics account and see/use the surveys.

**How to use the .qsv file:** Download the .qsf file of choice. Open your Qualtrics account (if accessing through Stanford account, you can login [here](https://uit.stanford.edu/service/survey)). Open a new or existing project. Click on Tools> Import/Export> Import Survey. Then the survey will appear as a project on your Home page.

For future studies, we suggest importing those files,making a copy of them, and editing the questions. That way, you wouldn’t have to rebuild the video portions of the survey (CrashCourse and CDC), which is the most time-consuming part.

=

For additional Qualtrics questions, contact **Lea** (9199492938, lea@teachaids.org).

**Vimeo uploading**

If a video needs to be embedded into the survey, Vimeo is a better platform to hold videos for a few reasons:

- Youtube is often blocked on many platforms
- Vimeo allows you to embed videos without title, video scrubber, and post-video suggestions

Upload onto the TeachAids vimeo account, with privacy setting at “Only people with a private link”.

Open up settings for each video, and click the “Embed” section. There should be a pre-set titled “Study”, which hides title, video scrubber, and post-video suggestions. If you do not see this pre-set or are using a different account for this video, all the settings to be tinkered with should be included in “Embed” and “Interaction” sections.

### Anticipating technical issues

Lea and I ran into a few issues where kids couldn’t move forward in qualtrics (more detail in [Tech issues](#_9g4p3clz674l)). To troubleshoot, we gave participants individual vimeo links and had them write down their survey questions (along with their names, etc) on paper.

If a concussion curriculum is composed of many videos (like the CDC Concussion in Sport video) it’s helpful to create a playlist. “Showcase” is Vimeo’s name for a playlist. In order to create a showcase with private videos on Vimeo (important for troubleshooting tech issues below), you need to change the privacy settings to make the playlist open only to people with a password. Otherwise, a public playlist will show as empty.

In the TeachAids Vimeo account, we have already created a private playlist of all CDC Concussions in Sports videos, with the password “study”.

Links for all the videos and/or “showcases” should be printed, along with paper copies of the survey questions.

For any Vimeo questions, contact **Angelina**.

## Travel Details

### Booking

**Flights**

Both flights to and from Denver were booked through Southwest. The airline allows you to reschedule flights without paying any penalties (you just pay the difference between the flights). For this trip, we had to reschedule to include an extra school. The number of students you actually get from each school will differ from the number the coach/AD gives you, so do be prepared to stay a few extra days to reach your minimum number. Southwest also allows two free checked bags (very important, as you’ll see from the packing list below).

**Accommodations**

Airbnbs are the way to go - they are typically more convenient, comfortable, and cheaper than hotels/hostels/motels.

Note: Outside of actually running the studies in schools, dropping of consent forms/gifts, etc, we spent a large amount of time working in our Airbnbs and nearby coffee shops. It’s important to choose a place that has adequate lighting, access to a fridge and kitchen, and a desk/table space for each RA.

**Car Rental**

Very expensive. We ended up having to pay much, much more than we were quoted, once all the applicable taxes and fees were applied. There is an **underage renter fee for all renters <25**. It’s important to look into your personal car insurance in advance - depending on the insurance plan, rental cars may or may not be covered to varying degrees. The TeachAids card covers insurance for all cars rented with it BUT as of July 2019, car rental companies require you to bring the card paying for the rental at pick-up.

Try to choose a car that has no windows into the trunk. Our trunk was always full (with water bottles, snacks, headphones, spare chargers), so it helps to ease the mind to have that all out of sight.

### Packing List

Personal items-

- Professional clothing
  - Note: If running the study in the summer, make sure you bring light clothing. Most of the schools we were at didn’t run air conditioning over the summer.
- Informal clothing for post-study afternoons
- Computers, chargers
- Phones, chargers, portable charge-banks
- Med pack - pain-killers, cold meds

Tech-

- Headphones (2x the number of kids at largest school - in case there is no time to disinfect between schools)
- Extra chargers for study participants’ personal laptops (likely Chromebook), extension cords
- Extra computers(?)
  - Note: We brought two extra TA computers, but never used them. All schools had sufficient computers/laptops for all participants

Paperwork-

- Manila envelopes for consent forms (2x school + extras)
- Envelopes for receipts (1x RA)
- Folders (1x study condition, plus an extra)
- Construction paper - same color as your tiny-url links

Misc Supplies-

- Lots of pens (for kids to sign student assent forms)
- Tape, scissors [IN CHECKED LUGGAGE], paperclips, rubber bands
- Gifts for coaches/athletic directors

Note: We brought ~600 sheets of paper back with us from the study location, and added 6 pounds to our luggage from consent forms alone. When packing before the study, make sure you leave extra room and/or bring an extra duffel bag for overflow.

### Upon Arrival - Buy

- Snacks - Nut free if possible (2x student)
- Water (1x student)
- Clorox wipes to disinfect headphones

### Upon Arrival - Print

We planned to borrow a printer from Piya’s family (ordered extra ink cartridges, set up a time to pick-up printer, etc), but unfortunately the printer was broken. We also realized that printing 1000 sheets of paper on a normal home printer would take many hours.

We ended up printing at FedEx (13 cents per page). It may alternatively be worth it to print in the office and bring them all with you in luggage.

- This document (1x RA)
- Parent permission slips (2x student)
- 18+ Student Consent forms (10x school)
- <18 Student Assent form (1x student + 20 extras per school)
- Survey URL printed largely in middle of page (for both survey conditions)
- Sheet of paper with 10 survey URLs (2x, for both survey conditions)
  - Cut these up, so you there are 20 small strips with survey URLs.
- Survey questions (50x school, only used as backup)
- Sheet of paper with all relevant vimeo links (10x school, only used as backup)

## Preparing for Each School

As mentioned in [Connecting with Schools and Coaches](#_pbktkucqyfo), it’s VERY IMPORTANT to have the coach’s phone number and be in contact in the weeks leading up to the study. Even if they do not respond to the texts, they do (mostly) read them. It’s best to try to get a meeting with coaches in person a week before to ask about number of participants, drop off parental consent forms, figure out the layout of the school, etc. If it’s not possible to meet with the coach, these details (especially the consent forms!!) should be hammered out through text/email/phone call.

### Randomization Sheet

The [randomization sheet](https://docs.google.com/spreadsheets/d/1qfdGsbFXeoNqd4Ay7n3GY-J1hs2EH2zwz_So053VFYo/edit?usp=drive_web&ouid=106040921074160714333) should be created before getting to the school. Create columns for First Name, Last Name, Parent Consent, Student Assent, Study Completed, Randomization (Preserved), and Randomization (Raw). The first five columns should be left empty, but you can pre-fill the randomization. In the first Randomization (Raw) cell [G2], type in the formula: =RANDBETWEEN(1,2) . If there are more than 2 study conditions, increase the second number as appropriate - ie. 3 study conditions: =RANDBETWEEN(1,3) . Drag down the formula to auto-populate more rows. Have randomized numbers for all anticipated study participants, plus a few extras. If we anticipated having 50 students, we auto-populated 60 or so.

Each time you make any edits to the page, the numbers will continue to change/randomize. Copy the numbers from the raw column, and paste values only (command-shift-v) into the Randomization (Preserved) column. The numbers will denote which group participants are in.

### To bring to each school

Tech-

- RA Computers, chargers
- Phones, portable charge-banks
- Headphones (1x student)
- Extra laptop chargers, Extension cords (if study participants using personal laptops)

Paperwork-

- Pens
- Manila envelopes
- 1 folder per study condition. In it:
  - This document
  - 18+ Student Consent forms (half the anticipated number of students + 10 extras)
  - <18 Student Assent forms (5)
  - Sign with relevant Qualtrics link
  - Survey questions (25, only used as backup)
  - Vimeo links (5, only used as backup)

Supples:

- Snacks (2x student)
- Water (1x student)

##

## At the schools

1. Text coach! “We’ve arrived, and are parking at [[location]].”

Note: If you need to register as visitors, OR if the Coach has not texted you back and you don’t have a pre-arranged location, enter the school building. There should be a front office – let them know you are there to meet the Coach, and that you are part of a Stanford research study. Be prepared to show or leave copies of your Stanford ID/Driver’s License/Other verifiable ID at the front desk in order to register as a visitor and gain access to the school campus.

1. Meet up with the coach/designated school contact. Thank them sincerely!!
2. Ask to be taken to classrooms. If applicable, ask for computer carts.
3. Ask coach for collected consent forms.
4. Ask where the coach will be during the study & where the team should go after they’re done w/ the survey.
5. **One RA:** Set up the space. Start setting up protein bars, stations for paperwork, etc

Try to set up two tables outside of the classrooms. One table should hold snacks and waters, and the other should be the check-in table. Try to position the tables so that kids can grab snacks before coming to check in, and then once they check in, you can indicate verbally and by hand gesture which room they’re supposed to be in.

If there is only one room, try to separate students by section of room. The most important thing is that kids with the same intervention aren’t sitting next to one another.

1. **Other RA:** Start typing student names (from parental consent forms) into randomization sheet.

### When Students begin Arriving

1. Inform them that they can get a snack and bottle of water
2. Ask them to line up and check in with an RA one by one.
3. Ask for their names, and use control-f function to find them in the form.

If their name is not in the sheet, ask if they have a parental consent form.

→ If they have it on hand, add their name into the sheet.

→ If they do not have one, politely tell them that we can not accept participants unless they have a consent form. Tell them to find their coach (the coach will likely have them run drills, etc).

1. Note that they are present on randomization sheet, and tell them **room assignment (or side of room assignment, if only one room).**
2. Give them **headphones** (if they don’t have their own)

## Conducting the Study

**Conduct a roll call!** Students will try to join their friends/get confused and go to the wrong room. Sort the randomization sheet by number, and read out only the kids that are supposed to be in your room. Tell them to say “here” and raise their hand when you call them.

Once all kids are where they should be, proceed with the script below.

### Script

*“Thank you for your participation in this study. My name is ____ - I’m one of the research assistants, and _____ is our assistant study coordinator. The purpose of the study is to examine concussion education. This is going to help millions of kids across the US, and it’s only made possible because of you!*

**<<Pass out student assent forms and pens>>**

*____ will be passing out an additional form for you to sign. It explains what you will be doing during this research session. In short, you will watch two concussion education curricula and answer three series of questions. Your participation in this study is entirely voluntary. Any questions?* ***- pause for questions -*** *We will give you a moment to read through and sign the assent form, and then ___ will collect it while I continue reading the directions.”*

***<<When it appears most students have signed, assistant study coordinator begins collecting forms and research assistant resumes with script>>***

*“I will continue with the directions now. This is not a test, but your opinions and your perspectives are so important to help better concussion education. It's important that you share your individual voices so please don't collaborate with others. Please be sure your phones are put away, if you have not put them away already.*

*After you’ve finished the survey, please raise your hand so we can ensure that the submission has gone through properly. After you’ve finished, you may use your phone, but we ask that you do not distract others or leave the room until everyone is finished. Any questions?”* ***- pause -***

*“We will now provide instructions for accessing the study materials. Thank you in advance for your patience. Please open Chrome and type in the URL that _____ is [[[holding up//writing on the board//passing out to you]]], which will automatically take you to the survey. However, please wait to begin completing the questions until after I start the timer.”*

*“Does everyone have the survey pulled up? Please raise your hand if you do not.* ***-pause -*** *Does everyone see a* ***[[[blue/orange - depending on survey condition]]]*** *box on the front page?”*

***<<assistant study coordinator can walk along the back of room to make sure everyone has link properly loaded>>***

*“Please plug in your headphones now. You may begin the study.”*

### Action items while students are taking study

- Ensure students are completing materials, not talking, etc
- Cross-check that all students marked “present” in your room have a collected student assent form
- Scan consent forms using CamScanner phone app (free, generates high quality PDFs)

Note: Stay in contact with each other throughout the study! Help each other troubleshoot/address any issues. Also, sometimes one group will be faster than the other, and it’s helpful for all RAs to know where each group is at in the process.

At the end of the study

- Thank students for completing the study
- Ask them to leave their headphones behind.

### If participants are being disruptive

- If there are two kids talking:
  - Walk over to stand by them and they’ll usually stop talking
  - If they continue, “Do you have a question?”
  - If they continue, “Please focus and try not to disrupt others. The survey will end soon and the faster everyone get through it, the faster they can leave.
- Students finishing the survey early-
  - Students can use their phones after they’ve finished their study, but can not be disruptive to other students
  - **Don’t let them leave until everyone is finished**

### Troubleshooting tech issues

Sometimes Qualtrics becomes finicky in the middle of the study - students aren’t able to move forward because 1) the video freezes or 2) the timer freezes and no blue forward button appears.

For the first time this occurs, just refresh the page. This will often fix the issue, and the student will be brought back to the same page (they will not have to retake the survey). If student expresses frustration that they already finished the video, reassure them that they will only have to rewatch the video once.

If it continues to occur, give the participant the link to video or playlist, and have them write out their answers on paper.

## After Study

- Clean up the space
- Disinfect headphones
- Send thank you notes (and gifts for extra helpful coaches/admin)

## Lessons Learned

**SCHEDULING/COACH COMMUNICATION**

**Start early -** especially if traveling for the study, allocate enough time to schedule the sessions with the schools in advance. In the Colorado 2019 study, we did not start recruiting early enough and faced a lot of stress and unexpected drop-outs at the last minute. If you are doing the study in the summer, you have to keep in mind that a lot of the staff is on vacation/holiday which might cause unexpected delays in scheduling.

**Meet in person & provide consent forms -** it was extremely helpful to meet the coaches at least once in person before the day of the study. They really appreciated it when we came by to leave blank consent forms and the schools where we did that were most prepared when we came back for the study. When we do not provide hard copies ourselves, we have to rely on the coaches to print and distribute them, which is a more uncertain approach.

**PREPARATIONS/MATERIALS**

**Extra earphones** - it is **essential** to bring single-use earphones. Usually, the teams come straight from practice and do not have earphones or headphones on them. In addition, make sure to **have enough extra pairs.** A large amount of the earphones end up not returned, lost or damaged so the quantity significantly decreases between sessions. Make sure to have enough extra pairs.

**Print with a professional printer ex. Fedex (if traveling) -** You will likely have to print hundreds of pages of parent and student consent forms. While it is tempting to want to save money by using a home printer in the hotel/Airbnb, it will be close to impossible to print the necessary amount of pages that way. Regular/home printers are too slow for the amount of fast bulk printing that is required to conduct a study with hundreds of participants. Make sure to use a professional printer, one that is equipped to deal with large orders, such as at a FedEx or another professional printer. While it costs more than using a personal printer, at least in our study, it saved an incredibly large amount of time (likely over 6h), and as with any other work trip, time was of the essence.

**Have an unlisted/private Vimeo playlist with the videos for each study condition -** There are many issues that could occur with Qualtrics, ex. YouTube is blocked or a certain page freezes and the student can’t progress with the rest of the survey. If that happens, it is good to have a backup of the videos in the form of a Vimeo (unlisted or private) playlist that you can access and provide to the student.

**Save videos in a Vimeo playlist (as back-up) -** There are many issues that could occur with Qualtrics, ex. YouTube is blocked or a certain page freezes and the student can’t progress with the rest of the survey. If that happens, it is good to have a backup of the videos in the form of a Vimeo (unlisted or private) playlist that you can access and provide to the student.

**CONDUCTING THE SESSIONS**

**Collect all parent consent forms before you allow students to come in the room -** It is essential to have everyone’s parent consent form before you collect any data. It is useful to set-up an “admin” table at the entrance of the room and have the RAs collect parent forms, randomize students and distribute students to the corresponding rooms. If you have a survey entry without a corresponding parent consent form, you will NOT be able to use the data and you will have to report the missing consent form in your paper.

**Collect all student assent forms before you the survey begins -** sometimes it is tempting to either 1) ask students to bring their assent forms when they finish the survey or 2) collect the forms when the students begin the surveys. **Neither of those is recommended** - make sure to collect the student assent forms **before** the surveys have begun. If you leave it for the end of the surveys, sometimes students leave before you have had the chance to check-in with them and collect the form. If you collect the student forms during the survey, the students get distracted and there is a lot of commotion. It is absolutely essential to have student assent forms for **all** participants - if you do not have a student assent form for a survey response, you can’t use the data entry and you will have to report the missing assent form in your paper.

**AFTER THE SESSION**

**If you can, save the consent/assent forms as separate files -**  It is understandable to scan them as one file if it is more convenient in the moment but if possible (especially if you have lots of RAs helping), scanning individually will significantly decrease the length and difficulty of post-study processing.
